# Proteomics Mapping of Cord Blood Identifies Haptoglobin “Switch-On” Pattern as Biomarker of Early-Onset Neonatal Sepsis in Preterm Newborns

**DOI:** 10.1371/journal.pone.0026111

**Published:** 2011-10-10

**Authors:** Catalin S. Buhimschi, Vineet Bhandari, Antonette T. Dulay, Unzila A. Nayeri, Sonya S. Abdel-Razeq, Christian M. Pettker, Stephen Thung, Guomao Zhao, Yiping W. Han, Matthew Bizzarro, Irina A. Buhimschi

**Affiliations:** 1 Department of Obstetrics, Gynecology and Reproductive Sciences, Yale University, School of Medicine, New Haven, Connecticut, United States of America; 2 Department of Pediatrics, Division of Perinatal Medicine Yale University, School of Medicine, New Haven, Connecticut, United States of America; 3 Department of Periodontics, Case Western Reserve University, School of Dental Medicine, Cleveland, Ohio, United States of America; University of Florida, United States of America

## Abstract

**Background:**

Intra-amniotic infection and/or inflammation (IAI) are important causes of preterm birth and early-onset neonatal sepsis (EONS). A prompt and accurate diagnosis of EONS is critical for improved neonatal outcomes. We sought to explore the cord blood proteome and identify biomarkers and functional protein networks characterizing EONS in preterm newborns.

**Methodology/Principal Findings:**

We studied a prospective cohort of 180 premature newborns delivered May 2004-September 2009. A proteomics discovery phase employing two-dimensional differential gel electrophoresis (2D-DIGE) and mass spectrometry identified 19 differentially-expressed proteins in cord blood of newborns with culture-confirmed EONS (n = 3) versus GA-matched controls (n = 3). Ontological classifications of the proteins included transfer/carrier, immunity/defense, protease/extracellular matrix. The 1^st^-level external validation conducted in the remaining 174 samples confirmed elevated haptoglobin and haptoglobin-related protein immunoreactivity (Hp&HpRP) in newborns with EONS (presumed and culture-confirmed) independent of GA at birth and birthweight (P<0.001). Western blot concurred in determining that EONS babies had conspicuous Hp&HpRP bands in cord blood (“switch-on pattern”) as opposed to non-EONS newborns who had near-absent “switch-off pattern” (*P*<0.001). Fetal Hp phenotype independently impacted Hp&HpRP. A Bayesian latent-class analysis (LCA) was further used for unbiased classification of all 180 cases based on probability of “antenatal IAI exposure” as latent variable. This was then subjected to 2^nd^-level validation against indicators of adverse short-term neonatal outcome. The optimal LCA algorithm combined Hp&HpRP switch pattern (most input), interleukin-6 and neonatal hematological indices yielding two non-overlapping newborn clusters with low (≤20%) versus high (≥70%) probability of IAI exposure. This approach reclassified ∼30% of clinical EONS diagnoses lowering the number needed to harm and increasing the odds ratios for several adverse outcomes including intra-ventricular hemorrhage.

**Conclusions/Significance:**

Antenatal exposure to IAI results in precocious switch-on of Hp&HpRP expression. As EONS biomarker, cord blood Hp&HpRP has potential to improve the selection of newborns for prompt and targeted treatment at birth.

## Introduction

Technological advances in newborn intensive care units have increased survival of preterm infants, and thus awareness of the need to improve outcomes [1]. Infection-related preterm birth (PTB) presents a unique clinical circumstance given the need to balance prolonging gestation against the risk of early-onset neonatal sepsis (EONS) and consequently of poor neonatal outcomes including respiratory distress syndrome (RDS), necrotizing enterocolitis (NEC), bronchopulmonary dysplasia (BPD), retinopathy of prematurity (ROP), intraventricular hemorrhage (IVH) and death, independent of gestational age (GA) at delivery [2]–[4].

Improving neonatal outcomes of premature infants has been hampered by poor prediction and identification of EONS during the antenatal and immediate postpartum periods [5]. With the exception of RDS, which manifests immediately after birth, most of the aforementioned neonatal complications manifest days (e.g. IVH) or even weeks post-delivery [6]. This suggests that the decision to treat EONS and administer preventive strategies is often taken late in the pathophysiologic process responsible for sepsis-related end-organ damage [6], [7].

Discovery of relevant biomarkers for better understanding of EONS and for improved identification of affected newborns is hypothetically possible through application of proteomics [2], [7]–[10]. The challenge, however, is to isolate the relevant biomarkers amongst the huge number of identified proteins [11]. To discover biomarkers characteristic of intra-amniotic infection and/or inflammation (IAI), our group devised a stepwise strategy based on sequentially applied mathematical filters [12]. We used surface-enhanced laser desorption ionization time-of-flight mass spectrometry and named our analysis method Mass Restricted (MR) scoring. Presence in amniotic fluid (AF) of at least 2 of the 4 discriminative biomarkers (defensin-2, defensin-1, S100A12, S100A8) was validated as diagnostic of IAI [12]. We further determined that presence of the S100A8 biomarker in AF of women with IAI was associated with EONS [2], [13]. Although the MR score enables antenatal identification of fetuses at risk for EONS, it has inherent limitations. AF is not easily obtainable nor does it provide direct insight upon functional protein networks relevant for EONS. Proteomics analysis of the cord blood (CB) offers the potential to close these gaps. The objective of this study was to perform a comprehensive analysis of the human CB proteome to discover biomarkers that broaden our pathophysiologic understanding of EONS and improve identification of affected newborns. Following our discovery phase, potential proteomic targets were validated in an independent cohort of premature neonates. Bayesian latent-class analysis (LCA) was further employed to construct an unbiased model to calculate the probability of “antenatal IAI exposure”, which was then validated against indicators of adverse short-term neonatal outcomes.

## Methods

### Ethics Statement

The Yale University Human Investigational Committee approved the study protocol. All mothers provided written informed consent.

### Study population and biological samples

**Figure 1:**
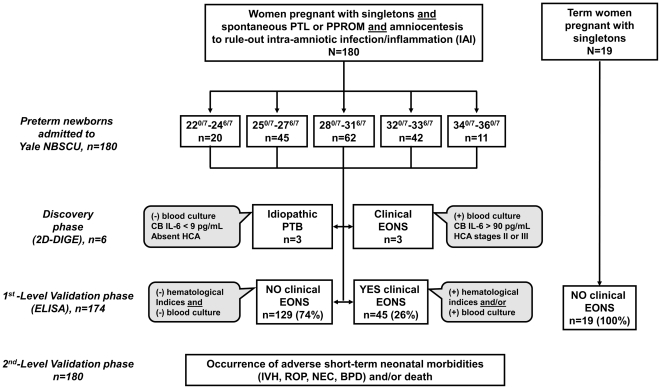
Flowchart representation of the stepwise study design and of the subject groups analyzed in each phase. Abbreviations: PTL, preterm labor; PTB, preterm birth; PPROM, preterm premature rupture of membranes; NBSCU, New Born Special Care Unit; CB, cord blood; EONS, early-onset neonatal sepsis; HCA, histological chorioamnionitis; IVH, intraventricular hemorrhage; ROP, retinopathy of prematurity; NEC, necrotizing enterocolitis, BPD, bronchopulmonary dysplasia; 2D-DIGE, two-dimensional differential-gel-electrophoresis.

Umbilical CB serum was retrieved from 180 consecutive preterm singleton newborns born to mothers who had an amniocentesis to rule out IAI (May 2004 to September 2009). All fetuses were live-born and admitted to the Newborn Special Care Unit (NBSCU) of Yale New Haven Hospital. In addition, paired maternal and CB samples were obtained from 19 healthy non-laboring women (GA: 38–40 weeks) undergoing elective cesarean delivery at term. A flowchart of newborns and samples analyzed during the discovery and validation phases is presented in Figure 1.

Preterm newborns were delivered by women presenting with symptoms of preterm labor, preterm premature rupture of membranes (PPROM), and/or advanced cervical dilatation (≥3 cm) [14]. Determination of GA was based on last menstrual period and ultrasound evaluation prior to 20 weeks [15]. Inclusion criteria: preterm singleton fetus born alive whose mothers had an amniocentesis to rule out intra-amniotic infection and inflammation (IAI), admission to Yale NBSCU, appropriate growth for GA, normal anatomic ultrasonographic survey. Exclusion criteria for maternal enrollment included: chronic medical conditions (*i.e.* hypertension, diabetes, thyroid disease), HIV, hepatitis or other viral infections, fetal structural abnormalities or fetal heart rate abnormalities requiring immediate intervention (i.e. bradycardia, or prolonged variable decelerations). Inclusion criteria for the term group: uncomplicated term pregnancy with indication for cesarean delivery for breech or prior uterine scar, appropriately grown fetus, reassuring fetal heart rate prior to surgery. Uterine contractions and/or cervical change consistent with labor were additional exclusion criteria for the term group.

Following amniocentesis each patient was followed prospectively to delivery, independent of our research protocol. In the absence of signs or symptoms of clinical chorioamnionitis (fever >37.8°C, uterine tenderness and/or fetal tachycardia), AF laboratory results suggestive of infection, non-reassuring fetal heart and/or abruption, PPROM was managed expectantly. Prior to delivery, corticosteroids for lung maturity and antibiotics were administered when clinically indicated [14]. Induction of labor or a surgical delivery was performed for indications such as AF laboratory results traditionally considered to indicate IAI, [2] prolapsed umbilical cord and/or GA ≥34 weeks [16]. The neonatology resuscitation team was present at the time of delivery for all newborns.

For clinical management, diagnosis of IAI was established based on well-recognized clinical, biochemical and microbiological AF tests which included: glucose concentration (cut-off of ≤15 mg/dL), lactate dehydrogenase activity (LDH, cut-off ≥419 U/L), white blood cell count (WBC, cut-off ≥50 cells/mm3), Gram stain and microbiological cultures for aerobes, anaerobes, *Ureaplasma* and *Mycoplasma* species [17], [18].

In all 180 cases, hematoxylin & eosin-stained sections of extraplacental membranes (amnion and chorio-decidua), chorionic plate and umbilical cord were examined systematically for inflammation. Three histological stages of chorioamnionitis [19] (stage I: intervillositis, stage II: chorionic inflammation, and stage III: full-thickness inflammation of both chorion and amnion) were complemented by a previously described histological grading system that includes 4 grades of inflammation of the amnion, chorio-decidua and umbilical cord [20].

Cord blood was retrieved by sterile puncture of the umbilical vein after delivery. Samples were centrifuged for 10 min (1,000g, 4°C) and the serum aliquoted and stored at −80°C.

### Diagnoses of EONS and of other short-term neonatal outcomes

Neonatal hematological indices were assessed from blood specimens obtained within 2-hours post-delivery. The diagnosis of EONS was “presumed” in the presence of at least two of the following hematological criteria: absolute neutrophil count of <7,500 or >14,500 cells/mm3; absolute band count >1,500 cells/mm3; immature/total neutrophil (I∶T) ratio >0.16; platelet count <150,000 cells/mm as previously described [13]. EONS was termed “confirmed” when neonatal blood cultures returned a positive result. “Clinical EONS” was defined as the presence of “presumed” EONS corroborated with clinical symptoms and/or “confirmed” EONS at ≤72 hours after birth. All newborns with clinical EONS received intravenous antibiotics in NBSCU.

Evaluation for intra-ventricular hemorrhage (IVH) was done per institutional protocol using serial cranial ultrasounds on days 3, 7–10 and 30 of life [6], [21]. Additional scans were performed if clinically indicated. The diagnosis and grading of IVH was established by experienced pediatric radiologists: grade 1, germinal matrix hemorrhage; grade 2, intraventricular blood without distension of the ventricular system; grade 3, blood filling and distending the ventricular system and grade 4, parenchymal involvement of hemorrhage, also known as periventricular venous infarction [21]. The ophthalmologist classified retinopathy of prematurity (ROP) in each eye according to the international classification [22]. Clinical, metabolic, hematologic and abdominal x-ray abnormalities (i.e pneumatosis intestinalis, portal venous gas) criteria were used to diagnose necrotizing enterocolitis (NEC) [23]. Bronhopulmonary dysplasia (BPD) was defined as need of receiving supplemental oxygen at 36 weeks' corrected postmenstrual age [24]. End of newborn follow-up was March 2010.

### Proteomics discovery phase

#### Characteristics of cases used for discovery

To identify biomarkers and functional protein networks characteristic of EONS, we first employed two-dimensional differential gel electrophoresis (2D-DIGE) on select CB samples. Three preterm newborns (GA: median, interquartile range [IQR]: 28 [25]–[30] weeks) had confirmed EONS by positive blood cultures for *Escherichia coli*. All had elevated fetal inflammatory status by CB interleukin-6 (IL-6) and their placentas had histological chorioamnionitis [25]. Three neonates without confirmed or presumed EONS, born at similar GA (26 [25]–[30] weeks) in the setting of idiopathic PTB (absent IAI, negative AF cultures, absent histological chorioamnionitis) were used as controls. There was no statistical GA difference between EONS and idiopathic PTB cases (paired t-test, *P* = 0.169).

#### 2D-DIGE protocol

Identification of low abundant proteins was facilitated with the aid of albumin and IgG removal kit (GE Healthcare, Piscataway, NJ), as previously described [26]. Cord blood proteins (50 µg) were labeled with either Cy5 (confirmed EONS group) or Cy3 (idiopathic PTB group). A reference pool (25 µg total protein) was labeled with Cy2 and used as internal control. For each pair, labeled samples were pooled and isoelectric focusing was performed on the Ettan-DIGE system (GE Healthcare, Piscataway, NJ) using an isoelectric point range of 3–10 followed by SDS-PAGE on a 12% gel for the second dimension. After spot detection, automatic background correction, spot volume normalization and volume ratio calculation, dye ratios were determined using DeCyder Extended Data Analysis software (GE Healthcare).

#### Protein identification and data mining

Spots corresponding to ≥1.5-fold changes were robotically excised using the Ettan Spot Picker instrument (GE Healthcare) and subjected to automated in-gel tryptic digestion on the Ettan TA Digester (GE Healthcare). Automated MALDI-MS/MS spectra were acquired on the 4800 TOF/TOF proteomics analyzer (Applied Biosystems, Foster City, CA). The resulting peptide sequences along with spot information (Cy5/Cy3 ratio, location on gels) were uploaded in the web-accessible Yale Protein Expression Database (YPED http://medicine.yale.edu/keck/proteomics/yped/index.aspx) [27]. A combined peptide mass fingerprint and tandem mass spectrometry search was done. Protein sequences were analyzed with Applied Biosystems GPS Explorer software and Mascot algorithm against the non-redundant National Center for Biotechnology Information (NCBInr) and the International Protein Index (IPI) databases [28]. A comprehensive list of probable identities (Mascot scores of ≥81 for NCBInr and ≥61 for IPI) was generated. Unique IPI identities differentially expressed ≥1.5 fold were classified using PANTHER (Protein Analysis through Evolutionary Relationships) (http://www.pantherdb.org). Identities were grouped into families and subfamilies of shared functions, and ontologically categorized by biological process, molecular function and pathway. A reductionist algorithm was applied to extract identities differentially expressed in at least two of the three 2D-DIGE gels. This approach pointed toward haptoglobin (Hp), haptoglobin-related protein (HpRP), α-fetoprotein (AFP), vitamin-D binding-protein (VDBP), apolipoprotein A4 (APOA4), apolipoprotein E (APOE) and apolipoprotein H (APOH) as potential CB biomarkers for EONS.

### 1^st^-Level validation phase

#### Immunoassays

The HpRP gene product has >90% sequence homology with Hp [29]. Identification of a distinct mRNA coding for HpRP continues to be elusive [30]. Furthermore, a specific antibody that discriminates HpRP from Hp does not exist. Thus, for the purpose of this study we refer to Hp&HpRP as the combined Hp and HpRP immunoreactivity measured with anti-Hp antibodies. Samples were diluted with blocking buffer (5% non-fat dry milk) 100, 1,000 or 10,000-fold (CB) or only 10,000-fold (maternal blood). A mixed phenotype Hp standard (Hp1-2, Sigma) was used to prepare a 7-point standard curve (250-3.9 ng/ml). Immunoassays for AFP (R&D Systems, Minneapolis, MN), VDBP (ICL Inc, Newberg, OR), actin-free VDBP (Alpco, Salem, NH), APOA4 (Cederlane Labs, Burlington, NC), APOE (MBL International, Woburn, MA and APOH (Enzyme Research Laboratories, South Bend, IN) were performed according to each procedure summary. In pilot experiments we determined the CB serum optimal sample dilutions for AFP (50,000-fold), VDBP (total 50,000-fold, actin-free 5,000-fold), APOA4 (100-fold), APOE (500-fold) and APOH (10,000-fold). IL-6 (Pierce-Endogen, Rockford, IL) was measured in AF and CB to assess the inflammatory status of the two biological compartments. All samples were tested in duplicate by investigators unaware of case origin or outcome. Cord serum total protein was quantified using bicinchoninic acid (BCA) assay (Pierce Biotechnology, Rockford, IL).

#### Hp&HpRP Western blotting

Haptoglobin is a tetrameric protein with two α and two β-chains linked by disulfide bonds. In humans, Hp occurs in two co-dominant allelic forms, *Hp1* and *Hp2*, which differ in the length of the α-chain [31]. The human population has 3 major Hp phenotypes (Hp1-1, Hp2-2 and the heterozygous Hp1-2), derived from variations in the α-chain with identical β-chains [29]. Absence of Hp at protein level denotes Hp0-0 phenotype (ahaptoglobinemia). Differences in Hp phenotypes can be distinguished by Western blotting.

SDS-PAGE gels (10-20%, InVitrogen, Carlsbad, CA) were loaded with equal amounts of CB protein (2 µg/lane) mixed 1∶2 with reducing sample buffer (Bio-Rad, La Jolla, CA) and boiled for 5 min. After electrophoretic transfer, nitrocellulose membranes (Bio-Rad) were blocked with 5% milk and then incubated overnight at 4°C rabbit anti-Hp polyclonal antibody (1∶3,000, Sigma, St Louis, MO). Detection was performed using biotinylated goat anti-rabbit secondary antibody (1∶5,000, Jackson Immunoresearch, West Grove, PA) followed by streptavidin-linked horseradish peroxidase, (1∶8,000, Amersham Biosystems, Piscataway, NJ), chemiluminescence (ECL-Plus, Amersham) and a timed 3 min. exposure to film (Kodak Biomax). Optical density of the bands of interest was analyzed with Image J software (NIH, http:\\ rsb.info.nih.gov). The results were expressed as the optical density of each band and as total optical density calculated from the sum of α- and β-chains. Purified Hp from blood of adults with known phenotypes (Hp1-1, Hp1-2 and Hp2-2, Sigma, St. Louis, MO) was used as positive control.

In the current study, a detectable Hp β-chain (∼42 kDa) on Western blot was indicative of a “switch-on” in Hp expression. Hp phenotypes were defined by additional presence of α-chain bands at either ∼9 kDa (α^1^: Hp1-1), ∼20 kDa (α^2^: Hp2-2) or both (Hp1-2). In select samples (n = 20) Hp phenotypes were determined both by the above western blot procedure and the classical peroxidase method [32]. We noted that our denaturing and reducing Western blot protocol was far more sensitive for detecting expressed Hp&HpRP patterns in CB compared to the peroxidase method which in our hands appeared suitable only for adult blood (data not shown).

### 2^nd^-Level validation phase

We applied latent-class analysis (LCA), a statistical solution that has gained increased acceptance when a perfect gold standard diagnostic test does not exist [33]. Briefly, LCA assumes that a hidden (latent) variable is responsible for heterogeneity among observed variables. By studying the patterns of co-variation among observed variables, the nature of the latent variable can be characterized. Specifically, rather than testing an *a priori* classifier (i.e. biomarker level above or lower than a pre-determined cut-off), the fit of a series of different models is examined. Each model combines several indicators (variables involved in cluster formation) and covariates (other demographical or clinical variables), potentially adding to cluster characterization. A cluster model is examined first, and then clusters are added until no further improvement in classification is observed. For the purpose of this study the latent variable was set as “*antenatal IAI exposure*”. This variable was then investigated for its ability to predict short-term adverse neonatal outcomes in comparison with the clinical diagnosis of EONS which represents the current standard of care. Since sepsis is a known ascendant of IVH, the 2^nd^-level validation phase was powered to detect differences in frequency of IVH with respect to clinical EONS.

### Statistical analyses

Normality testing was performed using the Shapiro-Wilk test. Data were compared with one-way ANOVA followed by Holm-Sidak method (parametric) or Kruskal-Wallis on ranks followed by Dunn's tests (non-parametric) as appropriate. Pearson correlations were used to measure co-linearity between the selected independent variables as well as other relevant relationships between dependent and independent variables. Comparisons between proportions were done with Chi-square tests. The phi-coefficient of correlation (Φ), an index of agreement for binary data was calculated. Stepwise multivariable regression analysis was used to determine concurrent relationships between variables and to correct for possible influences of GA and birthweight. Statistical analyses were performed with SigmaPlot 11.0 (Systat Software), MedCalc (Broekstraat, Belgium) and Latent Gold 4.5. (Statistical Innovations) softwares. A *P* <0.05 was considered statistically significant.

Goodness of fit of LCA models was evaluated using the Bayesian Information Criterion (BIC) and Akaike information criterion (AIC) [33]. Lower BICs indicate model improvement. Conditional bootstrap re-sampling procedures (500 random iterations) were employed to compare fit indices between models. The input of each indicator to the final model was measured by the nominal correlation coefficient Goodman-Kruskal tau-b (GK-θ). Finally, a posterior probability classifying each case to each cluster of the final model was calculated. Odds ratios (OR), positive likelihood ratio (+LR, number of times more likely that a newborn with a positive diagnosis will develop the adverse outcome compared to newborn with a negative diagnosis), negative likelihood ratio (-LR, number of times more likely that a newborn with a negative diagnosis will not develop the adverse outcome compared to newborn with a positive diagnosis) and number needed to harm (NNH, number of subjects needed to be exposed to a risk factor to cause harm in one subject that otherwise would not have been harmed) were computed to determine the risk of adverse neonatal outcomes. The lower the NNH the worse the risk factor.

## Results

### Differences in CB serum proteome of newborns with confirmed EONS

**Figure 2:**
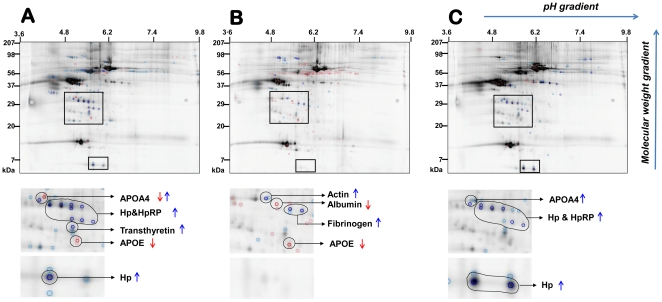
Merged 2D-DIGE images of the 3 gels used during the proteomics discovery phase and representative spots identified by mass spectrometry. Spots upregulated at least 1.5-fold are outlined in blue and spots down-regulated at least 1.5-fold are outlined with red. The squared regions are shown magnified below each gel (**A, B or C**) with matched unambiguous protein identities and direction of change represented by arrows. Apo, apolipoprotein; Hp, haptoglobin; HpRP, haptoglobin-related protein.

Mapping of CB proteomes identified 414 distinct protein spots on the three 2D-DIGE gels as shown in Figure 2. Cy5 (EONS)/Cy3 (idiopathic PTB) ratio was ≥1.5-fold different in 69 spots which were all subjected to identification by mass spectrometry. Sixty-seven spots matched at least one GenBank sequence identifier (down-regulated: 17 spots, up-regulated: 50 spots). With 1-5 identifiers/spot and multiple spots matching to the same identifier, our most inclusive list had 135 unique NCBInr identities. The analogous analysis against IPI human database found 70 unique IPI identities in at least 1/3 gels. Next, we excluded identifiers originating from a single 2D-DIGE gel with the rationale that these were most likely to result from randomness (technical or biological). This approach left us with 19 unambiguous, unique IPI human identities that changed in the context of EONS ≥1.5-fold in at least 2/3 gels. PANTHER further converged the 19 differentially expressed identities by matching several IPI identities to the same protein precursor as shown in Table S1.

**Figure 3:**
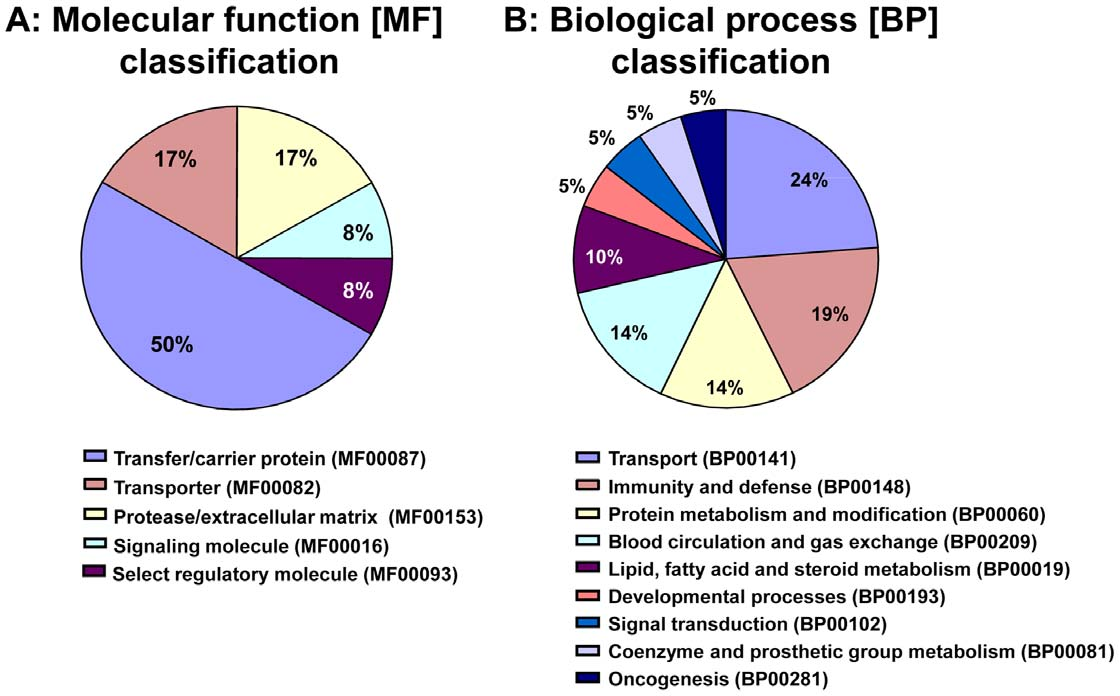
Pathway analysis results of the cord blood proteome dys-regulated in cord blood of premature newborns with culture confirmed EONS. Identities differentially expressed at least 1.5-fold were distributed by molecular function (**A**) and biological process (**B**) using PANTHER system.

Data mining using PANTHER distributed the 19 IPI identities into 5 distinct molecular functions and 9 biological processes with transport, immunity/defense and protease/extracellular matrix at the top of the list (Figure 3). Pending validation, this led us to conclude that CB of EONS newborns is potentially characterized by net up-regulation in HpRP (60-fold), Hp (33-fold), alpha-fetoprotein (7-fold), VDBP (2-fold) and by net down-regulation of albumin (6-fold), apolipoproteins APOA4 (5-fold), APOE (3-fold) and APOH (3-fold). Spots matching to α2-macroglobulin changed in divergent directions resulting in null net change.

### Results of 1^st^-level validation

#### Characteristics of cases used for 1^st^-level validation

The clinical characteristics of the mothers and newborns that provided the CB samples used in the 1^st^-level validation (n = 174) are included in Table S2. Results of clinical laboratory analyses are included in Table S3. Mothers of neonates with EONS were of lower GA at enrollment (amniocentesis), had shorter amniocentesis-to-delivery intervals and delivered at earlier GAs newborns with lower birthweights and lower Apgar scores. GA at delivery is an important determinant of neonatal maturity and outcome. Following correction for GA at birth we determined that EONS impacted independently on the 1-minute Apgar score. Women who delivered neonates with EONS more often had AF biochemical and microbiological tests results consistent with IAI. There was a higher frequency of histological chorioamnionitis and funisitis in the EONS group. Neonatal hematological tests and indices that were affected by EONS independent of GA were: neonatal WBC, neutrophil and lymphocyte counts, ABC and I∶T ratio.

Upon admission to NBSCU, 41 newborns were diagnosed with “presumed” EONS and placed promptly on i.v. antibiotics. In only 3 of these blood cultures returned a positive result. Blood cultures tested positive in another 4 newborns that did not show evidence of “presumed” EONS at admission. Thus, a total of 45 newborns from the 1^st^-level validation cohort had clinical diagnoses of EONS (excluding the 3 newborns used in the discovery phase). Together, these results point to the relevance of this cohort for external validation of potential CB biomarkers resulting from antenatal encounters with etiological agents of intra-amniotic infection, inflammation and EONS.

#### Immunoassay results

We chose to validate those proteomic targets with a net change ≥3-fold (HpRP, Hp, AFP, VDBP, APOA4, APOE and APOH). Despite albumin depletion prior to 2D-DIGE, we noted consistent changes in 12 spots matching to albumin (45-72kDa, Table S1). Although albumin variants and fragments thereof could be true biomarkers, specific knowledge on antibody affinity against each of the peptides is lacking at this time. Thus, changes in serum albumin were indirectly validated through the non-immunological measure of total protein concentration.

The results of validation immunoassays and of the additional relevant CB analytes (total protein and IL-6) are presented in Table 1. Cord serum Hp&HpRP immunoreactivity, but not that of the other proteomic targets, was significantly elevated in newborns with clinical EONS. Neonates with EONS had lower total CB protein and higher IL-6 levels. Following correction for GA at birth, only Hp&HpRP and IL-6 remained independently impacted by clinical EONS (GA-corrected levels *P* = 0.001 for both).

**Table 1:** Results of cord blood analytes measured in the 1^st^-level validation (n = 174).

Variable	NO EONS (n = 129)	YES EONS (n = 45)	*P* value
**Potential cord blood proteomic biomarkers**			
Hp&HpRP, *ng/mL* †	666 [17-6,818]	9,013 [4,449-12,463]	<0.001*
AFP, *µg/mL*	355 [213-606]	320 [154-466]	0.258
VDBP, total, *µg/mL*	381 [270-477]	438 [335-523]	0.145
VDBP, actin bound, *µg/mL*	205 [126-275]	232 [152-366]	0.132
VDBP, actin free, *µg/mL*	144 [112-193]	161 [118-203]	0.549
APOA4, *ng/mL*	2,373 [1,850-2,836]	2,143 [1,837-2,577]	0.338
APOE, *µg/mL*	284 [215-397]	331 [204-484]	0.404
APOH, *µg/mL*	137 [121-166]	145 [94-177]	0.950
**Other research analytes**			
Total protein, *mg/mL*	42 [37]-[49]	35.9 [31.6-43.6]	0.008
IL-6, *pg/mL*	8 [6]-[37]	106.5 [22.3-506.9]	<0.001*

† Data presented as median [interquartile range] and analyzed by Mann Whitney tests.

*Postnatal variables remaining significant for EONS after correction for GA at birth in multivariate analysis.

Abbreviations: EONS, early-onset neonatal sepsis; Hp&HpRP, combined haptoglobin and haptoglobin-related protein immunoreactivity; AFP, α-fetoprotein; VDBP, vitamin-D binding-protein; APOA4, apolipoprotein-A4; APOE, apolipoprotein-E; APOH, apolipoprotein-H; IL-6, interleukin-6.

#### Western blotting of CB Hp&HpRP

**Figure 4:**
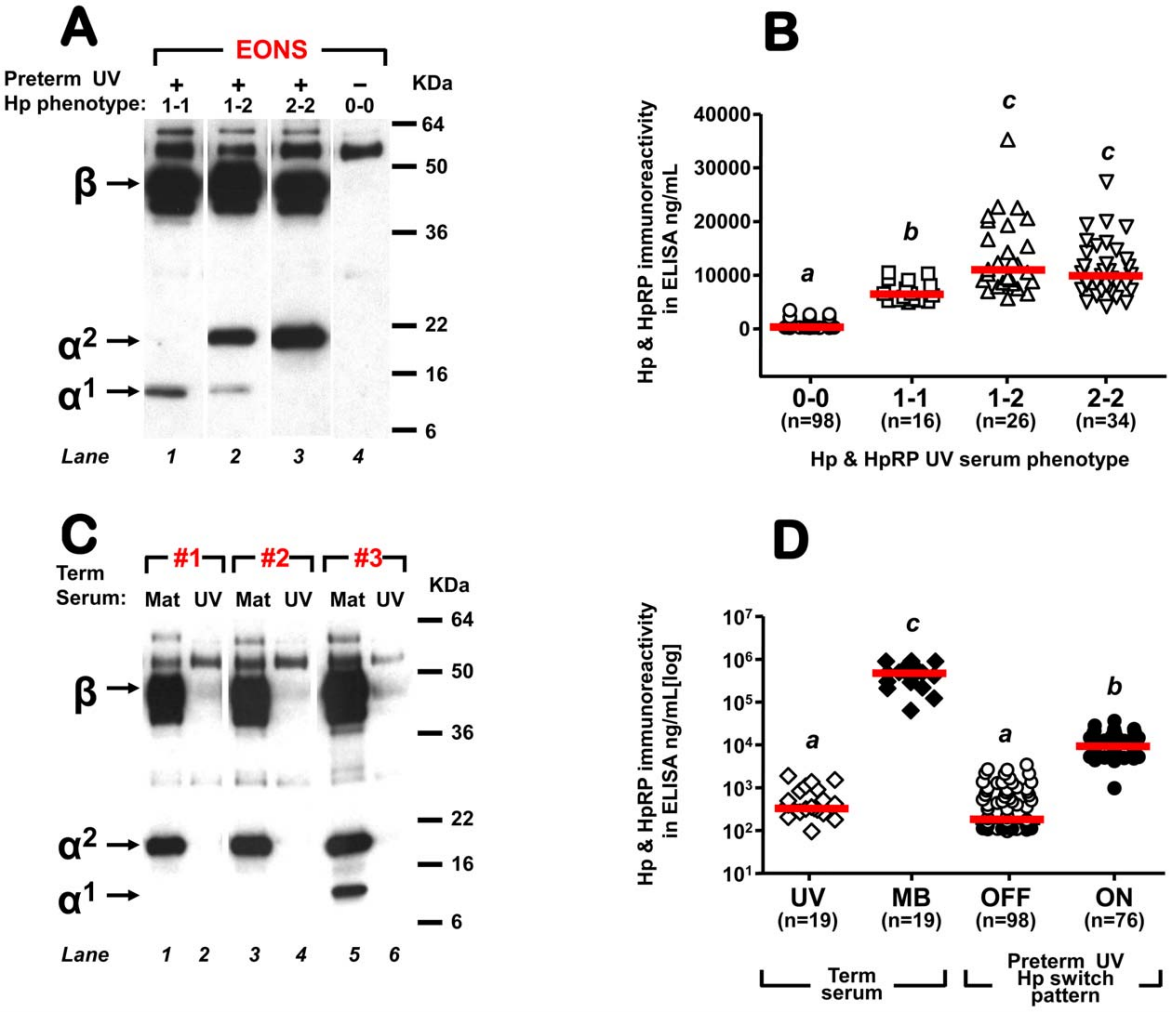
Cord blood haptoglobin (Hp) and haptoglobin-related protein (HpRp) immuno-reactivity revealed by Western blot (A&C) and ELISA (B&D) using antibodies reactive with both Hp and HpRP. (**A**) Western blot of umbilical vein (UV) serum from 4 preterm newborns of similar gestational age at birth. The newborns in *Lanes 1-3* were categorized as having early-onset sepsis (EONS) based on clinical manifestations and hematological indices and all received i.v. antibiotics. The newborn in *Lane 4* had a negative sepsis work-up. Blood cultures remained negative for all 4 newborns. The presence of a conspicuous immunoreactive band corresponding to the β-chain (∼42 kDa) in *Lanes 1-3* is consistent with our defined switched-on Hp pattern. The absence of this band indicates for a switched-off pattern in *Lane 4*. The band ∼9 kDa (*Lanes 1&2*) corresponds to the α1-chain whereas the band at ∼20 kDa (*Lanes 2-3*) corresponds to the α2-chain. Thus, the cord blood Hp patterns and phenotypes depicted in this gel are: switched-on pattern and Hp1-1 (*Lane 1*), switched-on pattern and Hp1-2 (*Lane 2*), switched-on pattern and Hp2-2 (*Lane 3*) and switched-off pattern and Hp0-0 (*Lane 4*). In our cohort, among preterm newborns with switched-on Hp pattern (present β-chain, n = 81), the distribution of Hp phenotypes was as follows: 6.0% Hp0-0 (5/81, no α-chain detected), 19.3% (16/81) Hp1-1, 32.5% (26/81) Hp1-2, and 42.1% (34/81) Hp2-2. The higher band intensity of the α2-chain compared to that of the α1-chain suggest that phenotype impacts on total Hp immunoreactivity. (**B**) Impact of Hp phenotypes on Hp&HpRP immunoreactivity as measured by ELISA. The red line indicates the group's median. Groups assigned different letters are statistically different at a *P*<0.05 (Kruskal-Wallis ANOVA). (**C**) Western blot of 3 representative maternal (Mat) and UV serum retrieved from women with normal deliveries at term. Note the switched-off pattern of the cord blood in contrast to the switched-on pattern of the adult blood. The Hp phenotypes of the mothers are Hp2-2 (Cases #1 and #2) and Hp1-2 in Case #3. (**D**) Quantitative comparison of Hp&HpRP immunoreactivity measured by ELISA in 19 UV and maternal blood (MB) serum from 19 normal deliveries at term relative to Hp&HpRP immunoreactivity measured in preterm UV with either switched-off or switched-on Hp pattern. The red line indicates the group's median. Groups assigned different letters are statistically different at a *P*<0.05 (Kruskal-Wallis ANOVA).

Figure 4A shows representative Western blots of CB serum of newborns with (lanes 1–3) and without (lane 4) clinical EONS. We found that a significantly higher number of EONS newborns displayed the Hp switched-on pattern at birth (Yes EONS: 78% (35/45) vs. No EONS 36% (46/129), *P*<0.001). A Hp switched-off pattern (Hp0-0) was characterized by significantly lower ELISA immunoreactivity compared to all other phenotypes (*P*<0.001, Figure 4B). Among newborns with switch-on pattern, the Hp phenotype impacted on ELISA immunoreactivity with Hp1-1 measuring lower compared to both Hp1-2 (*P*<0.001) and Hp2-2 (*P* = 0.002) (Figure 4B), independent of neonatal race, gender and GA at birth (*P* = 0.011).

To provide further evidence that precocious switch-on of Hp&HpRP is EONS-specific, we performed Western blotting and ELISA on matched CB and maternal blood from healthy gestations. Representative Western blot of 3 term maternal-fetal dyads are shown Figure 4C. No term baby (0/19) had switched-on Hp pattern (Hp0-0). This was in contrast to their mothers who all had detectable Hp chains (19/19), as expected for normal adult subjects. ELISA showed marked difference in Hp&HpRP between maternal and term cord serum with maternal exceeding CB concentrations by >3 orders of magnitude (*P*<0.001, Figure 4D). Preterm newborns with Hp switched-off pattern had similar Hp&HpRP to healthy term newborns (*P* = 0.189). Preterm newborns with switched-on pattern had significantly higher Hp&HpRP (*P*<0.001) compared to healthy and to preterm switched-off babies, albeit lower than maternal samples (*P*<0.001).

#### Considerations regarding determination of CB Hp&HpRP by ELISA and by Western blot

**Figure 5:**
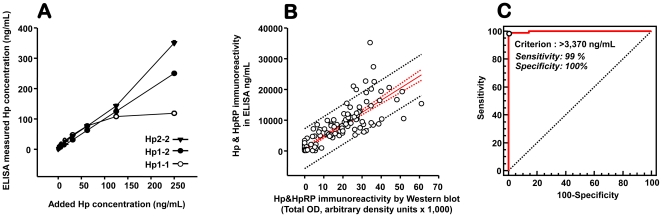
Considerations for determination of cord blood Hp&HpRP immunoreactivity. (**A**)Demonstration that Hp phenotype impacts on optical density (OD) measured in ELISA. Samples of adult Hp purified from individuals with known phenotype (Hp1-1, 1-2 or 2-2, purchased from Sigma) were diluted progressively in the range of the standard curve and analyzed in ELISA (n = 3 independent observations); (**B**) Relationship between cord blood Hp&HpRP immunoreactivity measured by ELISA and by Western blot in denatured and reducing conditions (n = 180). The continuous red line represents the linear regression line, the red dotted lines mark the 95% confidence interval and the dotted black lines the 95% prediction interval; (**C**) ROC analysis which demonstrates the relationship between Hp switch-on pattern and Hp&HpRP cord blood levels measured by ELISA. An ELISA cut-off of 3,370 ng/mL was deemed optimal to discriminate between the Hp switch-on and Hp switch-off pattern by Western blot (n = 180).

In Figure 5A we illustrate differences in optical density (OD) readings obtained with standard curves prepared with Hp purified from adult blood of different phenotypes: Hp1-1, 1-2 and 2-2. The same concentration of Hp1-1 measured less in ELISA compared to Hp1-2 and to Hp2-2 which rendered the highest values. This data suggests that different Hp phenotypes impact on Hp&HpRP ELISA levels and argues that reliance on ELISA level alone remains simplistic when assessing Hp&HpRP as CB biomarker.

There was a significant direct correlation between the total optical density of Hp&HpRP immunoreactivity by Western blot and by ELISA (R = 0.863, *P*<0.001, Figure 5B). A Hp&HpRP immunoreactivity >3,370 ng/mL in ELISA was best in indicating a switched-on Hp pattern in our preterm newborns (ROC area: 0.998, 95%CI [93.5–100], Figure 5C). This indicates that a binary indicator derived from ELISA immunoreactivity can potentially be used in lieu of Western blotting for determination of the Hp switch pattern. However, until isoform-specific antibodies are available, Western blotting may be an accessible method for concurrent determination of the Hp phenotype.

### Results of 2^nd^-level validation

#### Latent-class analysis (LCA) to identify the probability of antenatal IAI exposure

**Figure 6:**
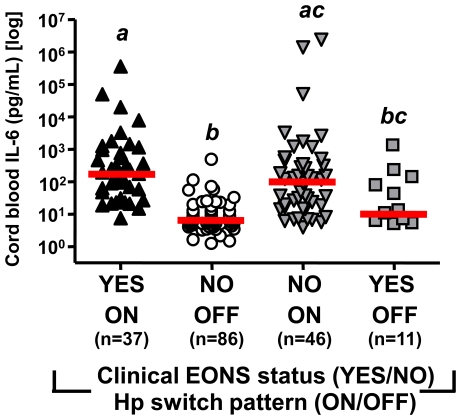
Cord blood interleukin-6 levels of preterm newborns in the study. Cases (n = 180) were stratified by clinical diagnosis of early-onset neonatal sepsis (EONS, Yes or No) and by haptoglobin (Hp) switch pattern (ON or OFF). The red line indicates the group's median. Groups sharing at least one common letter are statistically not different at a *P*>0.05 (Kruskal-Wallis ANOVA).

This analysis included all newborns in the study (n = 180). Preterm neonates who displayed the Hp switched-on pattern had significantly elevated CB IL-6 levels independent of EONS status (*P*<0.001, Figure 6). In multivariate analysis, CB IL-6 was independently impacted by the Hp switch-on pattern (*P*<0.001), number of expressed Hp2 alleles (none for Hp0-0 or Hp1-1, one for Hp1-2 and two for Hp2-2 phenotype; *P* = 0.005), and a clinical diagnosis of EONS (*P* = 0.003). Thus, chosen LCA discriminatory variables (indicators) were Hp switch pattern, IL-6 level and presumed EONS, as culture results are unavailable shortly after birth. Covariates were neonatal race, gender, Hp phenotype, GA and membrane status. In Table 2 we display the goodness of fit results for our 4 models. A decrease in the values of the Bayesian Information Criterion (BIC) indicates an improvement in the fit, with increasing the number of clusters. Generally speaking, models with *P*>0.05 provide an adequate fit (no significant difference between the model and the data). The model with the fewest number of parameters (most parsimonious) should be selected. Based on these criteria, Model 2 (2-cluster solution) appeared the best fit of our data. We next compared Model 2 with Model 3 using bootstrap statistical procedures. This analysis indicated that the improvement in classification rendered by Model 3 did not reach statistical significance (-2LL difference: 23, *P* = 0.07). Thus, Model 2 (the 2-cluster solution) was chosen as the best fit for our newborn population.

**Table 2:** Fit indices for possible latent-class analysis (LCA) models.

Model	# clusters	LL	BIC	AIC	Npar	L^2^	df	*P* value	Class Err
Model 1	1	-329.54	674.65	665.07	3	451.23	177	<0.001	0.000
Model 2*	2	-177.84	428.38	383.68	14	147.84	166	0.84	0.002
Model 3	3	-166.34	462.50	382.68	25	124.84	155	0.97	0.032
Model 4	4	-157.27	501.49	386.55	36	106.70	144	0.99	0.039

*Based on these considerations, Model 2 (the 2-cluster solution) was chosen as the optimal fit to our newborn population.

Abbreviations: **LL,** log-likelihood; **BIC,** Bayesian Information Criterion; **AIC,** Akaike Information Criterion. These statistical indicators weigh out the fit and parsimony by adjusting the LL to account for the number of parameters in the model. The lower the BIC and AIC values, the better the model. **Npar**, number of parameters; **L2**, likelihood-ratio goodness-of-fit value for the current model. **df,** degrees of freedom; **Class Err**, classification error. When classification of cases is based on modal assignment (to the class having the highest membership probability), the proportion of cases that are expected to be misclassified is reported by this statistical indicator. The closer this value is to 0 (zero) the better.

**Figure 7:**
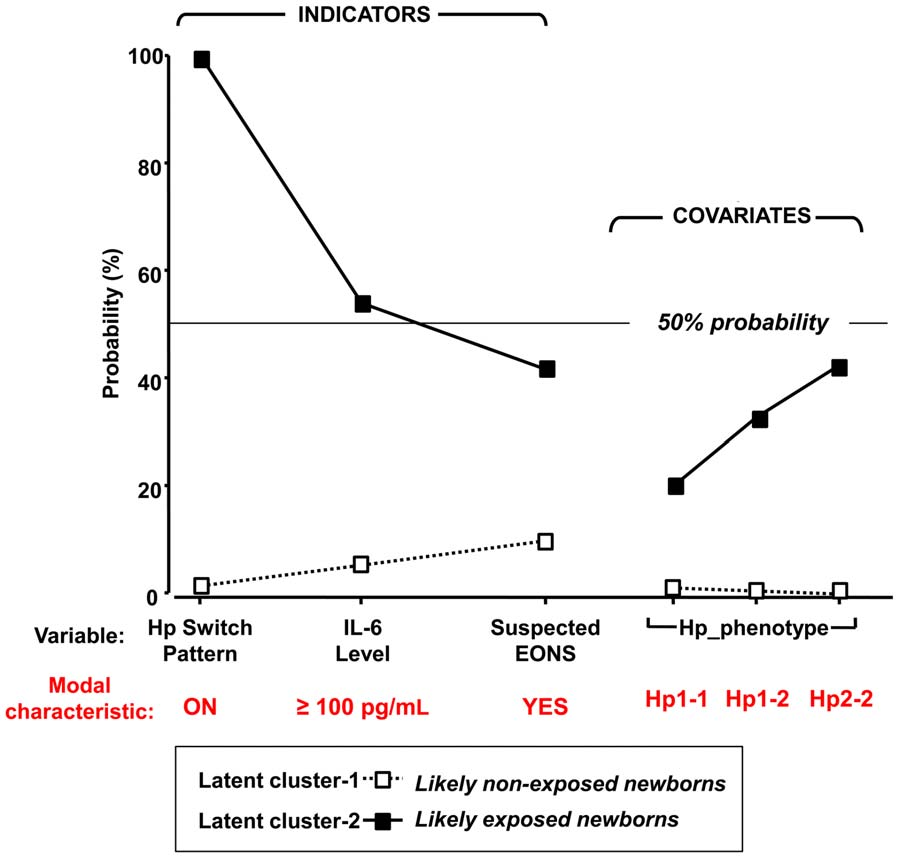
Results of Latent Cluster Analysis (LCA) applied to the population of preterm newborns in the study. (**A**) Profile plot of the 2-cluster model solution segregating the preterm newborn in this study (n = 180) by probability of antenatal exposure to intra-amniotic infection and/or inflammation (IAI). The *x-axis* lists the discriminative indicators and significant covariates with their modal characteristic (in red) for which the probability level expected to manifest in each of the two latent clusters is displayed on the *y-axis*. Newborns in *cluster-1* are characterized by low probability of a switch-on haptoglobin (Hp) pattern, IL-6≥100 pg/mL hematological indices consistent with presumed early-onset sepsis (EONS). In contrast, newborns in *cluster-2* are characterized by high probability of Hp switch-on pattern along with increased probabilities for cord blood IL-6≥100 pg/mL and for presumed EONS.

In our LCA model all 3 indicators contributed significantly (*P*<0.001), albeit unequally to data clustering (Table 3). Most of the input was achieved from Hp switching (GK-θ: 0.98). The least input was obtained from presumed EONS (GK-θ: 0.14). These results are graphically represented in Figure 7 which illustrates the non-overlapping characteristics of the two clusters. *Cluster-1* was characterized by very low probability for Hp switch-on pattern and low probabilities for presumed EONS and elevated CB IL-6 (>100 pg/mL). *Cluster-2* was characterized by a high probability for Hp switch-on pattern. Of the covariates, only the Hp phenotype showed statistical disparity between the two clusters (*P* = 0.002).

**Table 3:** Contribution of indicator variables in discriminating between latent clusters and partitioning of covariates between the clusters.

Variable	Variable levels	Wald *	*P* value	G-Kθ **
**Indicators**				
Hp&HpRP switch pattern	2 (ON or OFF)	14.88	<0.001	0.98
Cord blood IL-6 ≥100 pg/mL	2 (YES or No)	36.63	<0.001	0.30
Presumed EONS	2 (YES or NO)	21.32	<0.001	0.14
**Covariates**				
Hp phenotype	4 (Hp0-0, 1-1, 1-2, 2-2)	14.57	0.002	NA
Gender	2 (male or female)	0.40	0.530	NA
Race	2 (Caucasian or not)	1.97	0.160	NA
GA at delivery	2 (<30 wks or ≥30 wks)	1.00	0.320	NA
Membrane status	2 (PPROM or intact)	1.97	0.160	NA

*Wald statistic values are provided to assess the statistical significance of each nominal parameter. A non-significant associated *P* value means that the indicator does not discriminate between the clusters in a statistically significant way.

**G-Kθ (Goodman Kruskal tau b) is a more general coefficient of association between two nominal variables. In this case it represents the strength of association between the respective indicator and our latent variable “*antenatal IAI exposure*”. The closer to 1 the G-Kθ value, the higher the association and the contribution of the respective indicator in discriminating between latent clusters of the final model.

**Figure 8:**
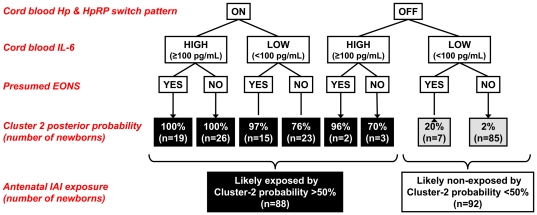
Clustering flowchart based on probability of “antenatal IAI exposure”. Posterior probabilities for inclusion in **cluster-2** were calculated for all 8 possible combinations of indicators (haptoglobin & haptoglobin [Hp&HpRP] switch pattern, cord blood IL-6, presumed EONS) and their modal characteristics. The number of newborns presenting each combination is included in parentheses. All newborns with *cluster-2* probability levels >50% (black boxes) were considered “likely exposed”. Alternatively, a diagnosis of “likely non-exposed” was assigned to newborns with *cluster-2* probability levels <50% (grey boxes).

Bayesian posterior probabilities of antenatal IAI exposure derived from the analysis of our study population are shown in a flowchart format in Figure 8. Each case was distributed to either *cluster-1* (“likely non-exposed”, n = 92) or *cluster-2* (“likely exposed”, n = 88) based on a posterior probability cut-off of 50% (equivalent to equally probable categories and a useless algorithm). Most of the input for the algorithm is derived from two CB indicators (Hp switch pattern and IL-6 ≥100 ng/mL). For the majority of cases (97%), the Hp switch pattern alone dictated the cluster assignment. Yet, 5 cases presenting with Hp switch-off pattern were solved by our model to *cluster-2* driven by the CB IL-6≥100 pg/mL. Although presumed EONS had the lowest discriminative power compared to the other LCA indicators and did not change the cluster assigned by the combination of the other two CB markers, it still contributed to the certainty level with which each case was assigned to each cluster.

The agreement between our novel classification and clinical EONS was “moderate” (Φ = 0.416, *P*<0.001). The cluster classification agreed with clinical diagnosis in only 69% (124/180) of cases. Our new algorithm identified a high probability of exposure in 27% (48/180) of cases deemed clinically EONS negative. In the remaining 4% (8/180) of cases, although these had been classified clinically as having EONS, our model argued against an exposed state. This suggests that based on current clinical practice, ∼30% of newborns are potentially misdiagnosed with respect to antenatal exposure to IAI. The results of cluster classification based on the three indicators particularized to the subgroup of newborns with positive blood cultures (n = 10), are presented in Table S4. Within this selected cohort, 20% (2/10) of newborns were found to be “likely non-exposed”. All the newborns were clustered as “likely exposed” although only 50% (4/8) had a diagnosis of presumed EONS with prompt initiation of antibiotic treatment.

#### Relationships between antenatal IAI exposure and short-term neonatal outcomes

**Figure 9:**
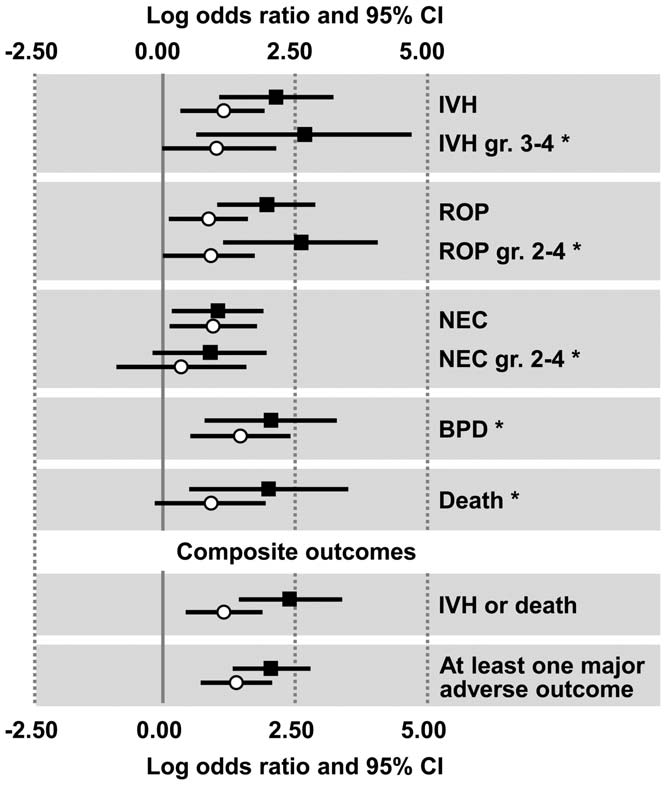
Results of 2^nd^-level validation against indicators of adverse neonatal outcome and impact of expressed Hp phenotype. (**A**) Forest plot illustrating the impact of newborn classification on prediction of neonatal complications (2^nd^-level validation). Open circles indicate the log odds ratios based on each newborn's clinical diagnosis of EONS. Closed squares indicate the log odds ratios based on assignment of newborns to either *cluster-1* or *cluster-2* based on a probability level of >50%. The length of each line shows the 95% confidence interval. The number of newborns affected by each adverse outcome and levels of statistical significance are included in Table 4. The asterisks (*) indicate major adverse outcomes taken into account in the composite variable.

Next we investigated the relationships between our new cluster classification and adverse neonatal outcomes and whether it could improve their prediction as compared to the current diagnosis of EONS. Our analysis was adequately powered to detect a relationship between clinical EONS and IVH as single outcome (power = 0.808, α = 0.05) and as part of the composite outcome IVH and death (power = 0.861). In multivariate logistic regression the two composite newborn outcomes were significantly predicted by the combination of GA (*P*<0.001), antenatal IAI exposure (*cluster-2* probability >50%, *P*<0.001) and the Hp phenotype (*P*<0.05). Excluded from the model were: CB IL-6, Hp&HpRP immunoreactivity by ELISA, presumed EONS and clinical diagnosis of EONS. As shown in Figure 9 and presented in more detail in Table 4, we determined that for all adverse outcomes with the exception of NEC, the ORs and +LR increased in the context of the new model. Using this algorithm, the +LR [95%CI] for the composite outcome “IVH or death” was 7.1 [2.9–17.4] versus 2.9 [1.7–5.0] for clinical EONS diagnosis. +LR for prediction of newborns developing “at least one major adverse outcome” was 4.3 [2.4–7.7] for our algorithm versus 2.8 [1.8–4.3] for clinical EONS. Negative LRs remained largely unchanged (composite outcome “IVH or death”, *cluster-2* inclusion: 0.65 [0.55–0.77] versus clinical EONS: 0.68 [0.54–0.87]; development of “at least one major adverse outcome”, *cluster-2* inclusion: 0.55 [0.44–0.70] versus clinical EONS: 0.53 [0.38–0.74]). Additionally, the lower NNH resulting from our classification suggests that inclusion in *cluster-2* (“likely exposed”) is a much stronger risk factor of neonatal morbidity than a diagnosis EONS in the context of current clinical practice.

**Table 4:** Comparison of case classification by probability of “antenatal IAI exposure” with clinical diagnosis of EONS for prediction of short-term adverse neonatal outcomes.

Adverse outcomes	Newborns with adverse outcome/total assessed	Classification by probability of “antenatal IAI exposure” (>50% Cluster-2)					Classification by clinical diagnosis of EONS				
		YES §	NO §	*P* value	OR [95% CI]	NNH	YES §	NO §	*P* value	OR [95% CI]	NNH
**Individual outcomes**											
IVH	30/170	26 (30%)	4 (5%)	<0.001	8.7 [2.9-26.2]	**2.3**	15 (31%)	15 (12%)	0.007	3.2 [1.4-7.3]	**3.8**
IVH gr. 3-4	14/170	13 (15%)	1 (1%)	0.003	14.8 [1.9-115.8]	**2.1**	7 (15%)	7 (6%)	0.114	2.8 [0.9-8.5]	**4.2**
ROP	37/169	31 (36%)	6 (7%)	<0.001	7.2 [2.8-18.5]	**2.4**	16 (33%)	21 (17%)	0.040	2.4 [1.1-5.1]	**5.3**
ROP gr. 2-4	24/169	22 (26%)	2 (2%)	<0.001	13.9 [3.2-61.4]	**2.1**	11 (23%)	13 (11%)	0.072	2.5 [1.0-6.0]	**4.9**
NEC	27/170	19 (22%)	8 (9%)	0.027	2.9 [1.2-7.0]	**4.0**	12 (25%)	15 (11%)	0.042	2.6 [1.1-6.1]	**4.8**
NEC gr. 2-4	16/170	11 (12%)	5 (5%)	0.161	2.5 [0.8-7.5]	**4.6**	8 (17%)	8 (6%)	0.055	1.4 [0.4-4.9]	**3.9**
BPD	20/167	17 (22%)	3 (3%)	<0.001	8.2 [2.3-29.3]	**2.3**	11 (26%)	9 (45%)	0.003	4.6 [1.7-12]	**2.9**
Death	15/180	13 (15%)	2 (2%)	0.005	7.8 [1.7-35.7]	**2.5**	7 (15%)	8 (6%)	0.127	2.6 [0.9-7.7]	**4.6**
**Composite outcomes**											
IVH or death	39/180	34 (39%)	5 (5%)	<0.001	11.0 [4.0-29.7]	**2.0**	18 (38%)	21 (16%)	<0.001	3.2 [1.5-6.7]	**4.0**
At least one major adverse outcome *	56/180	45 (51%)	11 (12%)	<0.001	7.7 [3.6-16.4]	**2.2**	26 (54%)	30 (23%)	<0.001	4.0 [2.0-8.1]	**3.2**

§ Data presented as n (%) and analyzed by Chi square tests.

Includes newborns with at least one of the following outcomes: IVH grade 3–4, ROP grade 2–4, NEC grade 2–4, BPD and/or death.

*Abbreviations: EONS, early-onset neonatal sepsis; IVH, intraventricular hemorrhage; ROP, retinopathy of prematurity; NEC, necrotizing enterocolitis; BPD, bronhopulmonary dysplasia; CI, confidence interval; OR, odds ratio; NNH, number needed to harm.

#### Relationships of Hp phenotypes with neonatal outcomes

**Figure 10:**
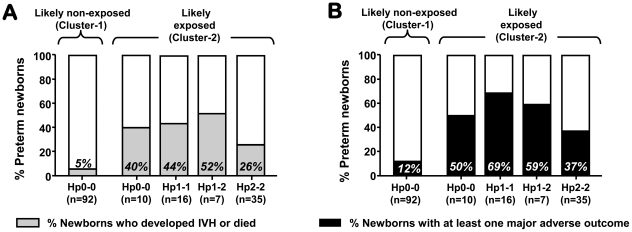
Relationships among cluster assignment, cord blood haptoglobin (Hp) phenotype at birth and composite outcome indicators. The percentages and the height of the grey or black portion of the bar indicate the proportion of newborns displaying the adverse composite outcome “IVH or death” (**A**) or “at least one major adverse outcome” (**B**). Abbreviations: IVH, intraventricular hemorrhage; ROP, retinopathy of prematurity; NEC, necrotizing enterocolitis; BPD, bronchopulmonary dysplasia; EONS, early-onset neonatal sepsis; IAI, intra-amniotic infection and/or inflammation.

Figure 10 demonstrates a disparity in adverse neonatal outcomes based on probability of antenatal IAI exposure and Hp phenotype. Among exposed newborns, those with Hp switched-on to Hp2-2 phenotype had the lowest incidence of IVH or death (Chi-square *P*<0.001) and of major morbidities (*P* = 0.043), despite no difference in GA at birth (*P* = 0.410).

## Discussion

There are several reasons why it is difficult to accurately diagnose EONS in current clinical practice. First, bloodstream infection in EONS fluctuates widely from 8% to 73% [34]. Many newborns with “presumed EONS” have nonspecific clinical manifestations (i.e lethargy, apnea, respiratory distress, hypoperfusion, shock), but negative cultures [35]. A second obstacle is technical and relates to the narrow spectrum of pathogens sought in microbiology laboratories. For example, searching for *Ureaplasma* and *Mycoplasma* spp. is not part of routine sepsis work-up in neonates. A study that evaluated the frequency of umbilical CB infections with these species found that that 23% of newborns born <32 weeks tested positive for these pathogens [36]. Moreover, it is plausible that analogous to AF infection, additional uncultivated and difficult-to-cultivate species could be also etiological agents of EONS [37].

Despite all the aforementioned obstacles, confirming or ruling-out EONS has important clinical decision-making consequences including prompt admission to NBSCU, antibiotic treatment, and resource allocation [38]. Each hour of delayed treatment worsens morbidity and mortality [39]. Forty-eight to 72 hours may pass until bacterial growth can be confirmed. Because even a 4-hour delay in initiation of antibiotics may increase neonatal mortality related to sepsis, universal “empirical antibiotic therapy” is advocated based on maternal risk factors and clinical suspicion of EONS [40], [41]. In most circumstances antibiotics are discontinued, if microbial culture results are negative, and neonatal clinical symptoms of sepsis absent [39]–[41]. In addition to the considerable financial burden, [42] there are significant downsides to this non-discriminatory therapeutic approach such as frequent monitoring of antibiotic blood levels, renal and oto-toxicity, increase risk of necrotizing enterocolitis (NEC) and changing the diversity of microbes in NBSCUs [39], [43], [44]. As a result, in the present state of clinical practice, hematological indices are the only laboratory aids available to the neonatologist with respect to clinical decisions to be made shortly after birth (i.e admission to NBSCU, intravenous antibiotic treatment and allocation of necessary resources) [13].

Attempts have been made to use physiologic parameters such as cytokine profiles (i.e. IL-6, IL-1β, IL-8 and TNF-α), non-specific acute-phase reactants (i.e. C-reactive protein, fibrinogen, amyloid protein A, procalcitonin), neutrophil CD64 marker, or PCR to identify neonates with EONS [45]–[48]. Unfortunately, clinical evidence suggests that no currently available test or combination of tests have sufficient sensitivity and specificity to ensure treatment of infected newborns, while allowing non-treatment of the non-infected infants. The time courses of many acute-phase reactants suggest that the serum level of many of these cytokines increase 6 to 8 hours after the onset of illness and peak after 2 to 3 days [49]. Therefore, their clinical usefulness might be limited for a targeted therapy immediately following birth. Hence, discovery of novel biomarkers able of revealing pathogenic pathways disturbed in fetuses exposed to infection while predicting the risk of EONS-related complications is critical.

We found that antenatal exposure to IAI in newborns with confirmed EONS affects major functional pathways including transfer/carrier, immunity/defense, and protease/extracellular matrix. Protein metabolism, blood circulation/gas exchange, lipid, fatty acid and steroid metabolism are additionally disturbed processes. Our proteomics discovery phase revealed alterations in several proteins largely synthesized by hepatic parenchymal cells (Hp, HpRP, AFP, VDBP, apopolipoproteins). Concerted changes in their expression patterns are considered signatures of hepatocellular reaction and/or damage [50]–[53]. Therefore, the fetal liver appears to be a key inflammatory target and an important control point for homeostatic adjustments in the context of IAI.

There is evidence to suggest that AFP has multiple cell growth regulating, differentiating and immunosuppressive activities [54], [55]. Besides serving as a transporter of vitamin-D, VDBP plays an important role in response to tissue injury and a variety of immune and inflammatory roles related to actin scavenging, leukocyte chemotaxis and macrophage activation [56]. Down-regulation of apolipoprotein's expression may be partially responsible for hyperlipidemia and increased triglyceride-rich lipoprotein levels observed in septic neonates [57]. This phenomenon may have biological significance as lipoproteins can bind LPS and protect against LPS-induced inflammation and injury [58]. Besides their down-regulation in inflammation, apolipoproteins may serve functions other than regulating lipid metabolism, such as inhibition of platelet aggregation and prostacyclin synthesis of the vascular endothelium [59], [60]. Recently, Ng et al. demonstrated that the apolipoprotein metabolism is dysregulated in neonates with late-onset sepsis and NEC [10]. Although the expression level of several apolipoproteins (ApoA1, ApoA2, ApoC2, ApoC3) was decreased, ApoC2 was the only non-redundant proteomic biomarker that appeared to have diagnostic value for the aforementioned clinical entities [10]. Developmental regulatory factors may impact on the fetal versus neonatal biologic adaptive response to IAI and may explain the differences the panel of dysregulated markers in cord versus neonatal blood.

Candidate proteomic targets are traditionally validated on alternative analytical platforms including ELISA. As shown, the immunoassay levels of several of our initial candidate biomarkers were not different between EONS and control groups. While this implies that several proteomic targets may not be clinically useful biomarkers when assessed through available immunoassays, involvement of the represented pathways in the pathophysiology of EONS should not be discredited. The apparent discrepancy is easily reconciled by technical aspects of both methods. ELISAs measure immunoreactivity of generic proteins, which, in EONS may undergo proteolysis. 2D-DIGE identifies the resulting fragments as distinct up- or down-regulated spots depending on the degree and sites of proteolysis. Yet, immunoreactive levels may not be impacted in parallel, as these largely depend on whether the proteolytic process affects the epitopes. In contrast to proteomics techniques, immunoassays evaluate proteins in native conditions with tertiary and quaternary structures independently impacting optical density [61]. In many instances, ELISAs do not discriminate among closely related proteins or among subunits of the same protein [61]. Our finding that the Hp&HpRP ELISA level sums the immunoreactivity of closely related proteins supports the complexity of this problem.

Hp is an acute-phase reactant glycoprotein primarily of hepatocytic origin [30]. Biological functions of Hp with potential roles in protecting the fetus and neonate from inflammation-derived injury include: scavenging of toxic hemoglobin, [62] dampening of neutrophilic oxidative burst, [63] inhibition of endotoxin-induced inflammation, suppression of monocytic TNF-α and IL-12 production, [64] and inhibition of B and T lymphocyte proliferation [65] IL-6 is a known transcriptional activator of Hp [66] and may explain why histological chorioamnionitis was associated with increased CB Hp levels [67]. It is believed that a gene located 2.2kb downstream to human Hp locus codes HpRP, [30] which in adult serum accounts for only ∼7% of total Hp&HpRP [68]. The α- and β-chains of HpRP are distinct, yet highly (∼90%) homologous with those of Hp, making separation of the two proteins difficult. A distinct role for HpRP in innate immunity was proposed recently in defense against trypanosome infection [69].

Our proteomic data complemented by ELISA and Western blotting provides methodologically unbiased evidence that exposure of the fetus to IAI leads to a precocious switch-on in Hp expression. These results resolve conflicting aspects in the literature regarding Hp. Following its description in 1938, [70] Hp was considered near-absent at birth switching to adult levels within the first year of life [71], [72]. We confirmed near-absent Hp in healthy term newborns. However, a few earlier studies reported assayable Hp in CB or neonatal blood, yet specifically noted that Hp level was not useful as marker of newborn sepsis [73]–[75]. The mix of term with preterm subjects, of early- and late-onset sepsis, inability to concurrently assess for IAI, reliance on less sensitive laboratory techniques optimal for assessing adult and nor cord blood Hp levels and on conventional statistics explain the discrepant conclusions among these reports, while supporting the novelty of our approach and findings.

The performance of a new diagnostic test is traditionally evaluated relative to a perfect “*gold standard”*. The current gold standard for infection, namely blood cultures, has well recognized limitations [76]. Clinical circumstances surrounding delivery (i.e. antenatal antibiotics, uncultivable bacteria as etiological agents, specimen contamination *ex-vivo*) increase the odds for EONS misclassification [37]. Our approach of using LCA was aimed to circumvent this obstacle [33], [77]. We established a diagnostic algorithm that adds two CB biochemical markers, (Hp&HpRP switch pattern and IL-6) to the hematological indices used for presumed EONS. When compared to clinical protocols for ascertaining EONS, our algorithm improved the predictive odds for postnatal complications, such as IVH which is known to have antenatal antecendants related to IAI [78]. Yet, Hp&HpRP switching exerted the highest discriminative power. When switched-on at birth, this biomarker alone resulted in probability of IAI of >70%.

An important regulator of Hp expression is the inflammatory cytokine IL-6 [66]. Hepatic Hp synthesis is dependent on cis-acting elements localized within the first 186 bp of the 5′-flanking region of the promoter [66]. Interaction of this promoter site with trans-acting elements is postulated to provide a 2-nd level of complexity in regulation of Hp expression. These observations may explain why individuals with the same Hp genotype, the levels of Hp vary with exposure to environmental or epigenetic stressors (physical effort, methylation status) [79]. Few individuals may lack both *Hp* alleles giving raise to Hp0-0 phenotype. In the US adult population, ahaptoglobinemia varies from 0.1% in Caucasians to much higher frequency 4% in African-Americans. Studies in knock-out mice have demonstrated that ahaptoglobinemia is associated with higher malarial parasitic burden consistent with Hp's beneficial role in protecting against infection [80].

In our study, the addition of CB IL-6 to the Hp switch pattern was useful only to solve as as “likely exposed” a minority of cases (∼3%, 5/180) that paradoxically retained the Hp switch-off pattern despite elevated CB IL-6 levels (range 110–1,533 pg/mL). One of these cases had confirmed EONS. Following our validation phase and LCA analysis, it became apparent that these cases represented ahaptoglobinemic outliers. We found that newborns retaining the Hp0-0 phenotype despite antenatal IAI exposure had an increased frequency of adverse outcomes consistent with the important anti-inflammatory and anti-microbial roles of Hp [64]. Thus, functional hypo- or ahaptoglobinemia may increase susceptibility to infection, inflammation and cell damage. Although in our study, the number of newborns with this phenotype (high CB IL-6 and switch-off Hp pattern) was likely too small for LCA to identify a third cluster, our algorithm permits isolation of this subgroup of newborns for future genetic and promoter kinetics studies.

The predictive value of Hp&HpRP switching as biomarker of antenatal IAI exposure was independent of GA and of its absolute concentration, which was phenotype-dependent. The disparity in Hp phenotypes among individuals has been studied extensively in relationship to susceptibility to infection as well as chronic diseases with oxidative component [81]. It has been suggested that individuals of Hp1-1 phenotype are able to handle free hemoglobin more efficiently but also to have reduced antibody responses due to the greater ability of Hp1-1 to inhibit lymphocyte transformation. In contrast, Hp2-2 individuals tend to have higher antibody responses and relative protection against infection at the expense of higher incidence of autoimmune conditions. Macrophages activated by the polymeric Hp2-2, shift the T-helper cell response toward Th-1 cytokines (IL-2, interferon-γ, lymphotoxins), while those activated by Hp1-1 synthesize more Th-2 cytokines (IL-4, IL-5, IL-6, IL-10) [82]. Our finding that Hp2-2 newborns appear to have better outcomes independent of prematurity is provocative and concurs with the idea that the Hp2 allele may offer survival advantage for this population.

In summary, we carried out a proteomics study to identify CB biomarkers of EONS and found that a switch-on in Hp&HpRP expression reflected a fetal adaptive response to IAI exposure *in utero*. Assessment of CB Hp&HpRP switch pattern carries the potential for quick turn-around and may provide useful information for neonatal risk stratification even in the absence of amniocentesis or placental histology. We propose that this biomarker could be a useful addition to the diagnostic work-up of EONS to increase the level of confidence that the newborn is at high risk of prematurity-related complications.

## Supporting Information

Table S1 **2D-DIGE and PANTHER results with convergence of unambiguous differentially expressed identities into proteomics targets.**
(PDF)

Table S2 **Clinical characteristics of the mothers and newborns who provided cord blood used during the 1st-level validation (n = 174).**
(PDF)

Table S3 **Laboratory characteristics of the mothers and newborns who provided cord blood used during the 1st-level validation (n = 174).**
(PDF)

Table S4 **Results** **and cluster assignment for the newborns with positive blood culture (confirmed EONS, n = 10).**
(PDF)
