# Supplementary material for: Proteomics Mapping of Cord Blood Identifies Haptoglobin “Switch-On” Pattern as Biomarker of Early-Onset Neonatal Sepsis in Preterm Newborns
Source: PLoS One. 2011 Oct 10;6(10):e26111. doi: 10.1371/journal.pone.0026111 (PMC3189953; doi:10.1371/journal.pone.0026111)
Supplement: Table S1 — 2D-DIGE and PANTHER results with convergence of unambiguous differentially expressed identities into proteomics targets. (PDF) [file pone.0026111.s001.pdf]

Table S1. 2D-DIGE and PANTHER results with convergence of unambiguous differentially expressed identities into proteomics targets

IPI ID	Spot MW (kDa)	Avg Spot PI	Avg DB score	Avg ion score	% coverage	# Spots UP	# Spots DOWN	Protein precursor	SwissProt Accession	#Gels	Avg fold UP	Avg fold DOWN	Net change
**NET CHANGE UP-REGULATED**													
IPI00477597	38.9	6.4	118	91	26	7	0	**HpRP**	**P00739**	**2 of 3**	**60**	**0**	**60 fold up**
IPI00607707	43.0	6.4	114	91	24	7	0						
IPI00641737	46.7	6.3	419	345	42	18	0	**Hp**	**P00738**	**2 of 3**	**33**	**0**	**33 fold up**
IPI00478493	38.4	6.1	426	344	50	18	0						
IPI00431645	31.4	8.5	480	376	66	15	0						
IPI00022443	68.6	5.5	393	270	54	5	0	**AFP**	**P02771**	**2 of 3**	**7**	**0**	**7 fold up**
IPI00742696	52.9	5.3	732	622	56	1	1	**VDBP**	**P02774**	**2 of 3**	**8**	**-6**	**2 fold up**
**NET CHANGE DOWN-REGULATED**													
IPI00022434	71.7	6.3	765	590	67	2	9	**ALB**	**P02768**	**3 of 3**	**4**	**-10**	**6 fold down**
IPI00745872	69.3	5.9	708	544	64	3	9						
IPI00878517	56.2	6.8	642	517	64	2	9						
IPI00384697	47.3	6.0	531	430	62	2	9						
IPI00216773	45.1	5.8	390	289	68	1	7						
IPI00304273	45.4	5.3	382	189	79	2	1	**APOA4**	**P06727**	**2 of 3**	**9**	**-4**	**5 fold down**
IPI00847179	45.3	5.3	350	172	75	2	1						
IPI00021842	36.1	5.7	841	576	120	0	2	**APOE**	**P02649**	**2 of 3**	**0**	**-3**	**3 fold down**
IPI00878953	32.5	7.0	591	421	101	0	2						
IPI00879456	24.9	5.8	448	292	109	0	2						
IPI00298828	38.3	8.3	482	406	58	4	4	**APOH**	**P02749**	**2 of 3**	**3**	**-6**	**3 fold down**
**NO NET CHANGE**													
IPI00478003	163.2	6.0	765	572	37	3	1	**A2MG**	**P01023**	**2 of 3**	**2**	**-2**	**zero**

Abbreviations: IPI, International Protein Index; Avg, average; DB , database; HpRP, haptoglobin-related protein; Hp, haptoglobin; AFP, α-fetoprotein; VDBP, vitamin-D binding-protein; ALB, albumin; APOA4, apolipoprotein-A4; APOE, apolipoprotein-E; APOH, apolipoprotein-H; A2MG; A2MG, α-microglobulin.
