# Supplementary material for: Proteomics Mapping of Cord Blood Identifies Haptoglobin “Switch-On” Pattern as Biomarker of Early-Onset Neonatal Sepsis in Preterm Newborns
Source: PLoS One. 2011 Oct 10;6(10):e26111. doi: 10.1371/journal.pone.0026111 (PMC3189953; doi:10.1371/journal.pone.0026111)
Supplement: Table S2 — Clinical characteristics of the mothers and newborns who provided cord blood used during the 1st-level validation (n = 174). (PDF) [file pone.0026111.s002.pdf]

Table S2. Clinical characteristics of the mothers and newborns who provided cord blood used during the 1st-level validation (n=174)

Variable	CLINICAL EONS		*P* value
	NO (n=129)	YES (n=45)	
**Maternal demographic and clinical characteristics at enrollment (amniocentesis)**			
Maternal age, *years* †	30 [23-34]	26 [21-34]	0.157
Gravidity †	2 [1-4]	3 [2-4]	0.394
Parity †	1 [0-2]	1 [0-2]	0.884
Race §			0.090
Caucasian	58 (45)	11 (24)	
African-American	44 (34)	19 (42)	
Hispanic	20 (16)	12 (27)	
Other	7 (5)	3 (7)	
Non-Caucasian race §	71 (55)	34 (76)	0.025
Gestational age, *weeks* †	29 [26-32]	26 [25-29]	<0.001
Cervical dilation, *cm* †	1 [0-3]	3 [1-4]	0.001
Ruptured membranes §	81 (63)	25 (56)	0.497
Uterine contractions §	56 (43)	20 (44)	0.957
History of preterm birth §	33 (26)	18 (40)	0.101
Clinical chorioamnionitis §	11 (9)	5 (11)	0.828
**Antenatal drug treatments/exposure**			
Steroids §	119 (92)	45 (100)	0.121
Antibiotics §	107 (83)	39 (87)	0.727
Tocolysis §	53 (41)	22 (49)	0.462
Magnesium sulfate §	42 (32)	20 (44)	0.210
Progesterone during pregnancy §	18 (14)	10 (22)	0.287
**Pregnancy outcome characteristics**			
Amniocentesis-to-delivery, *hours* †	24 [8-111]	8 [5-20]	0.003
Indicated preterm delivery §	66 (51)	31 (69)	0.059
Gestational age at delivery, *weeks* †	30 [28-33]	26 [25-30]	<0.001
Birthweight, *grams* †	1,450 [1,090-1,985]	920 [770-1,517]	<0.001
Cesarean delivery §	58 (45)	14 (33)	0.236
Apgar score at 1 minute †	8 [5-9]	5 [3-7]	<0.001*
Apgar score at 5 minutes †	9 [8-9]	8 [6-8]	<0.001
1 min Apgar <7 §	42 (32)	29 (64)	<0.001
5 min Apgar <7 §	14 (10)	16 (36)	<0.001
Newborn male gender §	62 (48)	20 (44)	0.806

† Data presented as median [interquartile range] and analyzed by Mann Whitney tests.
§ Data presented as n (%) and analyzed by Chi-square tests.
*Postnatal variable remaining significant for EONS after correction for GA at birth in multivariate analysis.
