# Supplementary material for: Proteomics Mapping of Cord Blood Identifies Haptoglobin “Switch-On” Pattern as Biomarker of Early-Onset Neonatal Sepsis in Preterm Newborns
Source: PLoS One. 2011 Oct 10;6(10):e26111. doi: 10.1371/journal.pone.0026111 (PMC3189953; doi:10.1371/journal.pone.0026111)
Supplement: Table S3 — Laboratory characteristics of the mothers and newborns who provided cord blood used during the 1st-level validation (n = 174). (PDF) [file pone.0026111.s003.pdf]

Table S3. Laboratory characteristics of the mothers and newborns who provided cord blood used during the 1st-level validation (n=174)

Variable	CLINICAL EONS		*P* value
	NO (n=129)	YES (n=45)	
**Amniotic fluid analysis**			
Glucose, *mg/dL* †	19[10-32]	3 [2-13]	<0.001
LDH, *U/L* †	260 [158-583]	744 [270-2,365]	<0.001
WBC, *cells/mm³* †	24 [4-540]	444 [59-1,840]	<0.001
Positive Gram stain §	27 (21)	31 (69)	<0.001
Positive microbial cultures §	28 (30)	36 (80)	<0.001
**Umbilical cord blood analysis**			
Arterial pH †	7.31 [7.28-7.34]	7.32 [7.25-7.35]	0.972
Arterial base deficit †	4.4 [3.2-5.5	6.1 [3.5-9.4]	0.074
Venous pH	7.36 [7.33-7.39]	7.38 [7.28-7.40]	0.440
Venous base deficit	3.8 [2.2-5.1]	4.8 [2.4-8.0]	0.143
Arterial or Venous pH <7 §	1 (1)	2 (6)	0.287
**Placental histological analysis**			
Chorionic plate inflammation, *stages II-III* §	60 (48)	41 (91)	<0.001*
Amnionitis, *stages 2-4* §	46 (36)	37 (82)	<0.001*
Choriodeciduitis, *stages 2-4* §	84 (66)	42 (93)	<0.001*
Funisitis, *stages 1-4* §	45 (35)	30 (67)	<0.001*
**Neonatal sepsis work-up**			
WBC, *cells x 1,000/mm³* †	10 [8-14]	15 [9.3-21]	0.003*
Hemoglobin, *mg/dL* †	15.1 [13.8-16.2]	14 [12.6-15.4]	0.004
Hematocrit, *%* †	46.1 [41.7-50.5]	43.6 [39.2-47.9]	0.014
Platelets, *cells x 1,000/mm³* †	269 [222-308]	236 [176-331]	0.118
Segmented neutrophils, *%* †	36 [25-44]	27 [19-39]	0.010*
Immature neutrophils (bands), *%* †	2 [0-6]	17 [13-25]	<0.001*
Lymphocytes, *%* †	44 [32-57]	29 [22-45]	<0.001*
Monocytes, *%* †	11 [7-15]	10 [8-15]	0.901
Absolute neutrophil count (ANC), *cells/mm³*	3,550 [2,164-6,166]	4,028 [2,148-6,778]	0.688
Absolute band count (ABC), *cells/mm³*	255 [0-674]	2,560 [1,456-3,808]	<0.001*
Immature/total neutrophil (I:T) ratio, %	2 [0-6]	17 [13-25]	<0.001*
Presumed EONS §	0 (0)	41 (91)	<0.001*
Culture positive (confirmed) EONS §	0 (0)	7 (16)	<0.001*

† Data presented as median [interquartile range] and analyzed by Mann Whitney tests.
§ Data presented as n (%) and analyzed by Chi-square tests.
*Postnatal variable remaining significant for EONS after correction for GA at birth in multivariate analysis.
