# Supplementary material for: Proteomics Mapping of Cord Blood Identifies Haptoglobin “Switch-On” Pattern as Biomarker of Early-Onset Neonatal Sepsis in Preterm Newborns
Source: PLoS One. 2011 Oct 10;6(10):e26111. doi: 10.1371/journal.pone.0026111 (PMC3189953; doi:10.1371/journal.pone.0026111)
Supplement: Table S4 — Results and cluster assignment for the newborns with positive blood culture (confirmed EONS, n = 10). (PDF) [file pone.0026111.s004.pdf]

Table S4. Results and cluster assignment for the newborns with positive blood culture (confirmed EONS, n=10)

Case #	GA at delivery (wks)	CB Hp Switch pattern	CB IL-6 (pg/mL)	CB IL6 >100 (pg/mL)	Presumed EONS	Cluster-2 probability (%)	Likely Exposed	Cultivated microorganisms
1*	28 1/7	OFF	77	NO	YES	20	NO	*Staphylococcus* spp<br>*Corynebacterium* spp
2	26 1/7	ON	49,835	YES	YES	100	YES	*Streptococcus* Group B
3	24 1/7	ON	19,818	YES	YES	100	YES	*Escherichia coli*
4	30 2/7	OFF	1,533	YES	NO	70	YES	*Escherichia coli*
5*	26 2/7	OFF	6	NO	NO	2	NO	*Staphylococcus* spp
6	25 1/7	ON	90	NO	YES	97	YES	*Escherichia coli*
7	24 4/7	ON	461	YES	YES	100	YES	*Escherichia coli*
8	25 0/7	ON	354,860	YES	NO	100	YES	*Escherichia coli*
9	25 5/7	ON	3,252	YES	NO	100	YES	*Streptococcus* Group B
10	24 6/7	ON	7,744	YES	NO	100	YES	*Streptococcus* Group B

* Cases 1 & 5 were classified as “likely non-exposed” by our algorithm despite the fact that blood cultures returned a positive result. The nature of the cultivated microorganism argues for *ex vivo* contamination of the specimen rather than EONS. The remaining cases were all classified as “likely exposed” despite infection with either Gram positive (n=3) or Gram negative species (n=5).
